# Fluoro-Modulated Molecular Geometry in Diketopyrrolopyrrole-Based Low-Bandgap Copolymers for Tuning the Photovoltaic Performance

**DOI:** 10.3389/fchem.2019.00333

**Published:** 2019-05-15

**Authors:** Cai'e Zhang, Yahui Liu, Jia Tu, Shouli Ming, Xinjun Xu, Zhishan Bo

**Affiliations:** Beijing Key Laboratory of Energy Conversion and Storage Materials, College of Chemistry, Beijing Normal University, Beijing, China

**Keywords:** diketopyrrolopyrrole, fluorination, supramolecular interaction, polymer solar cell, photovoltaic performance

## Abstract

Fluorination of conjugated polymers is an effective strategy to tune the energy levels for obtaining high power conversion efficiency (PCE) in organic solar cells. In this work, we have developed fluoro-modulated molecular geometries in diketopyrrolopyrrole based low-bandgap copolymers. In these polymers, planar conformation can be locked by intramolecular non-covalent interaction (intramolecular supramolecular interaction) between the sulfur atoms and the introduced F atoms (F···S interaction). By varying the fluorinated moieties, such a planarity can be disturbed and the molecular geometry is tuned. As a result, the polymer' properties can be modulated, including the ultraviolet-visible absorption spectrum to become broaden, charge mobility to be enhanced, open-circuit voltage (*V*_oc_) and short-circuited current (*J*_sc_) to be elevated, and thus photovoltaic performance to be improved. The photovoltaic device based on PCFB, one of the fluorinated terpolymers, exhibited a high PCE near 8.5% with simultaneously enhanced *V*_oc_ and *J*_sc_ relative to the non-fluorinated one (PCB).

## Introduction

Low-band gap (LBG) polymers have attracted lots of research attention due to their possibility to extend the absorption of solar spectrum from ultraviolet-visible (UV-vis) to near-infrared (NIR) region to make a good utilization of solar energy when used in organic solar cells (OSCs). They have the merits of enhancing the light absorption, realizing high-efficiency tandem solar cells with a wide bandgap polymer (Dou et al., 2012; You et al., 2013), and achieving semitransparent photovoltaic devices that strongly absorb light in the NIR region while allowing most of the visible light to get through (Dou et al., 2013). Among these LBG polymers, conjugated polymers employing diketopyrrolopyrrole (DPP) unit were frequently reported in the areas of organic field-effect transistors (OFETs) and OSCs in recent years (Hendriks et al., 2014a; Li et al., 2014b, 2018; Li C. et al., 2016; Yu et al., 2016). The DPP moiety usually has strong intermolecular interactions in the solid state including hydrogen bonding and π-π interactions and its polar nature enhances the tendency of DPP-based polymers to crystallize (Sonar et al., 2010). As a result, DPP-based conjugated polymers often exhibit an advantageously tunable and broad optical absorption, high charge carrier mobilities, and good nanoscale morphologies (Nielsen et al., 2013; Li et al., 2015; Wang et al., 2016), which can result in high photocurrents and good fill factors (FF) in OSCs. For instance, the optical absorption of the DPP based polymers can extend much farther into the NIR region (Hendriks et al., 2014b; Li et al., 2015), which provides wide photo-response in a long wavelength range to give a high short-circuit current (*J*_sc_) in OSCs and can be applied in NIR organic photodetectors (Ashraf et al., 2015).

However, there are also some drawbacks in DPP based OSCs. Their open circuit voltage (*V*_oc_) was usually <0.7 V due to their shallow highest occupied molecular orbital (HOMO) energy levels. Therefore, there are only several reports showed a power conversion efficiency (PCE) over 8% in single-junction OSCs based on DPP polymers (Hendriks et al., 2013; Choi et al., 2015; Zheng et al., 2016). There are several ways to adjust the energy level of polymers. One approach is to introduce electron-withdrawing groups in the backbone of the polymers. For example, poly (2,5-bis(2-decyltetradecyl )-pyrrolo[3,4-c]pyrrole-1,4(2H,5H)-dione-3,6-diyl-alt-3″,4′-difluoro-2,2′:5′,2″:5″,2′″-quaterthiophene-5,5′″-diyl (PDPP4T-2F) was successfully designed by introducing two fluorine atoms to the 2,2′-bithiophene monomer which gave deeper energy level and higher *V*_oc_ (Zheng et al., 2016). Introducing F atom is also important in tailoring the chemical and physical properties of the resulting polymers (Albrecht et al., 2012; Li et al., 2014a). The non-covalent interaction of F atom not only exists in copolymers, but also can be observed in non-fullerene small molecule acceptors (Zhao et al., 2017; Liu et al., 2018). Because fluorine is the most electronegative element in the periodic table, the F atom is a powerful functional group for donor/acceptor materials. It has a quite strong electron-withdrawing nature, so the introduction of fluorine into the polymer/acceptor backbone can lower the HOMO and the lowest unoccupied molecular orbital (LUMO) energy level, resulting in the increase of *V*_oc_. It is also believed that the fluorine atom offers non-covalent attractive interactions in a molecule (i.e., intramolecular supramolecular interaction) between the hydrogen or sulfur atoms, which may contribute to enhancing the coplanarity of the polymer backbone which would be favorable for the self-assembly and the crystallinity of the polymer (Li et al., 2014a; Kawashima et al., 2016; Zhang Q. et al., 2017). Another way to adjust the energy levels of polymers is to fabricate copolymers with different units. Recently, terpolymers which comprise three various components in the backbone have emerged as a new design strategy for donor polymers (Qin et al., 2014; Duan et al., 2016; Wang X. et al., 2017; Wang Y. et al., 2017; Huo et al., 2018). Among these copolymers, regioregular ones provide natural advantages, such as well-defined molecular structure, highly reproducibility and better molecular packing (Qin et al., 2014; Kim et al., 2015; Lee et al., 2015). Based on the above thinking, DPP based regioregular terpolymer with difluorobenzene and difluorocarbazole units was successfully fabricated and applied as donor material for OSCs in our previous work with a high *V*_oc_ of 0.86 V and PCE over 8% (Liu et al., 2016).

Despite such advantages of F substitutions to the properties of donor polymers, some reports demonstrated that excessive F atoms would reduce the photovoltaic performance due to the significantly aggravated aggregation of polymer chains and enhanced trap-assisted charge recombinations (Jo et al., 2015; Kawashima et al., 2016; Lee et al., 2016). It should be noted that such results were all obtained based on donor-acceptor (D-A) alternating binary copolymers. However, investigations of the effect of fluorine substitutions on both LBG terpolymers and DPP based copolymers are all currently absent. How the F atoms will affect the properties of such copolymers and their photovoltaic performance? With these questions in mind, here we expect to investigate the photovoltaic performance of DPP-based terpolymers by introducing fluorine atoms into different moieties of the polymer backbone and adjusting the number of fluorine atoms so as to modulate their properties. In this work, four polymers (**PFCFB, PCFB, PFCB**, and **PCB**) were designed by altering F atoms in the monomers. They were synthesized with Suzuki coupling and exhibited similar molecular weight. Their HOMO energy levels were significantly reduced with the introduction of F atoms which gave higher *V*_oc_ in the photovoltaic device. F atoms was able to flatten the adjacent aromatic units because of the F···S and F···H interactions. The F···S and F···H interactions are ultimately electrostatic interaction. In the previous literature (Fei et al., 2015), according to density functional theory (DFT) calculations, positively charged sulfur atom (+0.308) and negatively charged fluorine atom (−0.281) tend to form strong electrostatic interaction. However, hydrogen atom (+0.148) is less positively charged than sulfur atom, so F···S rather than F···H intramolecular interaction is preferred in our polymer backbone. Besides, Yan et al obtained single crystal structure of the model compounds (Li Z. et al., 2016). The observations of F···S interactions dominate the conformation or geometry. Therefore, we think the backbone of our polymer tend to form F···S intramolecular interaction. In addition, the FF was decreased with the diminution of F atom. The photovoltaic device based on PFCFB exhibited the highest PCE of 8.48% with a highest *V*_oc_ of 0.86 V and the device based on PCFB displayed a similar PCE of 8.46% with the highest *J*_sc_ of 17.20 mA cm^−2^, which are all improved relative to that of devices based on the non-fluorinated polymer (**PCB**).

## Results and Discussion

**Scheme 1:**
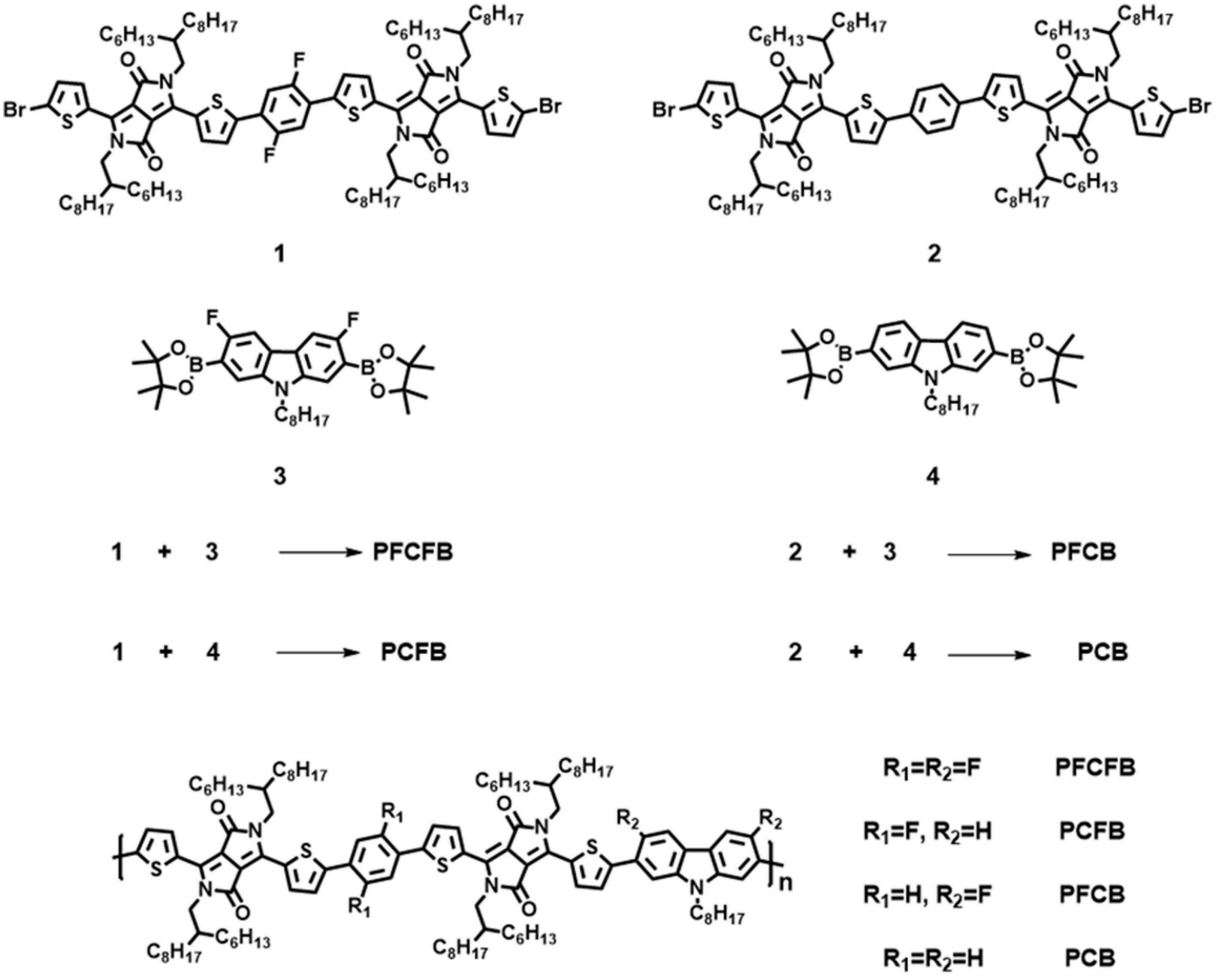
Molecular structures and synthetic methods of the polymers.

The synthetic route of polymers **PFCFB, PCFB, PFCB**, and **PCB** was shown in Scheme 1 (see Table S1). The original monomers **1**, **2**, **3**, and **4** were synthesized as reported (Du et al., 2013; Park et al., 2013; Liu et al., 2016). These polymers can be acquired by Suzuki coupling using Pd(PPh_3_)_4_ as the catalyst. The molecular weights of these polymers via gel permeation chromatography (GPC) measurements are listed in Table 1. It is worth noting that high number-average molecular weight over 250 kg/mol can be acquired with facile Suzuki polymerization for these polymers.

**Table 1:** The physical, optical, and electrical properties of polymers.

**Polymer**	***M*_**n**_ (kg/mol)**	***M*_**w**_ (kg/mol)**	**PDI**	**λ_**max**_ (nm) in film**	***E*_**g, opt**_ (eV)**	**HOMO (eV)**	**LUMO (eV)**
PFCFB	264	728	2.8	403, 677, 738	1.57	−5.31	−3.74
PCFB	284	1,006	3.5	393, 678, 742	1.55	−5.29	−3.74
PFCB	285	886	3.1	403, 670, 733	1.57	−5.28	−3.71
PCB	278	923	3.3	387, 667, 728	1.60	−5.25	−3.65

**Figure 1:**
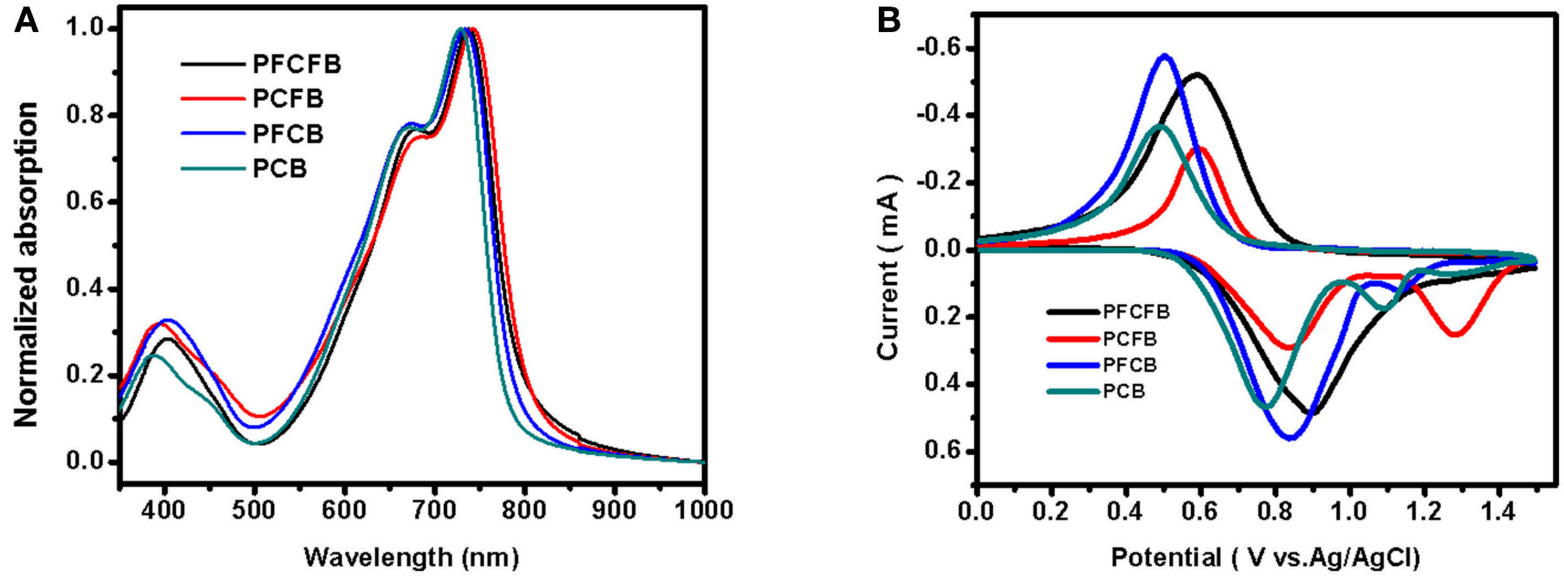
UV-vis spectra of polymers as thin films **(A)**, and CV curves of polymer films on a Pt electrode measured in 0.1 M Bu_4_NPF_6_ acetonitrile solutions for oxidation at a scan rate of 100 mV/s **(B)**.

UV-vis spectra of polymers were investigated in thin films (Figure 1A). These polymers show similar absorption in visible light region with two absorption bands. The one located at shorter wavelength can be attributed to the localized π-π^*^ transition and the strong peaks located at longer wavelength originate from the intramolecular charge transfer (ICT) (Tanaka et al., 2013). The absorption edges of these polymers are not the same. Optical bandgaps (*E*_g_) calculated from the absorption edges of these polymers are listed in Table 1. It shows that fluorination of the polymer backbone reduces the optical bandgap. In addition, the dominant absorption peak of **PCFB** (~740 nm) is slightly red-shifted compared with that of other polymers. This result can be explained by the shorter lamellar distance existing in **PCFB** film as validated from X-ray diffraction (XRD) data (*vide infra*, see Figure S1, Table S2), which indicates that the polymer chains were packed more tightly (Wang Y. et al., 2017). The electrochemical properties were also investigated by cyclic voltammetry (CV). Based on the CV results, energy levels of these polymers can be calculated according to the equations: *E*_HOMO_ = -*e*[*E*_ox, onset_ – *E*_(Fc/Fc+)_ + 4.8] (Li G. et al., 2016; Zhang C. E. et al., 2017), and *E*_LUMO_ = *E*_HOMO_ + *E*_g, opt_, where *E*_g, opt_ is the optical bandgap of the polymer. The detailed information is shown in Figure 1B, Table 1. As expected, the HOMO energy level was tunable and lowered in an observable scale with the introduction of F atoms, which is consistent with literature (Liu et al., 2018). Inevitably, the *V*_oc_ of corresponding devices is affected, which will be discussed further below. The thermostability was investigated with thermogravimetric analysis (TGA) shown in Figure S2 which indicates good thermal stability with a decomposition temperature over 350°C with a weight loss of 5% under nitrogen atmosphere. The packing behaviors of **PFCFB**, **PCFB**, **PFCB**, and **PCB** in films was surveyed by XRD measurements. All of these polymers exhibit two diffraction peaks. As displayed in Figure S1, Table S2, one set of the diffraction peaks in **PFCFB**, **PCFB**, **PFCB**, and **PCB** films are located at 2θ of 4.76°, 4.84°, 4.75°, and 4.79°, corresponding to lamellar distances of 18.58, 18.26, 18.61, and 18.44 Å, respectively. Diffraction peaks arising from the π-π stacking of backbones appeared at 2θ of 24.03°, 23.85°, 23.92°, and 23.63°, corresponding to distances of 3.70, 3.73, 3.72, and 3.77 Å for **PFCFB**, **PCFB**, **PFCB** and **PCB**, respectively. The consequence indicates that **PCFB** with fluorinated benzene but non-fluorinated carbazole moieties in the polymer chain possesses the minimum lamellar distance, while **PFCFB** with both fluorinated benzene and fluorinated carbazole moieties shows the minimum π-π stacking distance. Since the long-wavelength absorption peak (700~750 nm) is susceptible to the aggregation of polymer chains and the short-wavelength one (around 400 nm) is influenced by the π-π stacking of aromatic units, the XRD results are thus in line with the UV-vis absorption spectra of the polymers, where **PCFB** shows the most bathochromic-shifted peak in the long-wavelength region but **PFCFB** does so in the short-wavelength one.

## Density Functional Theory Calculations

**Figure 2:**
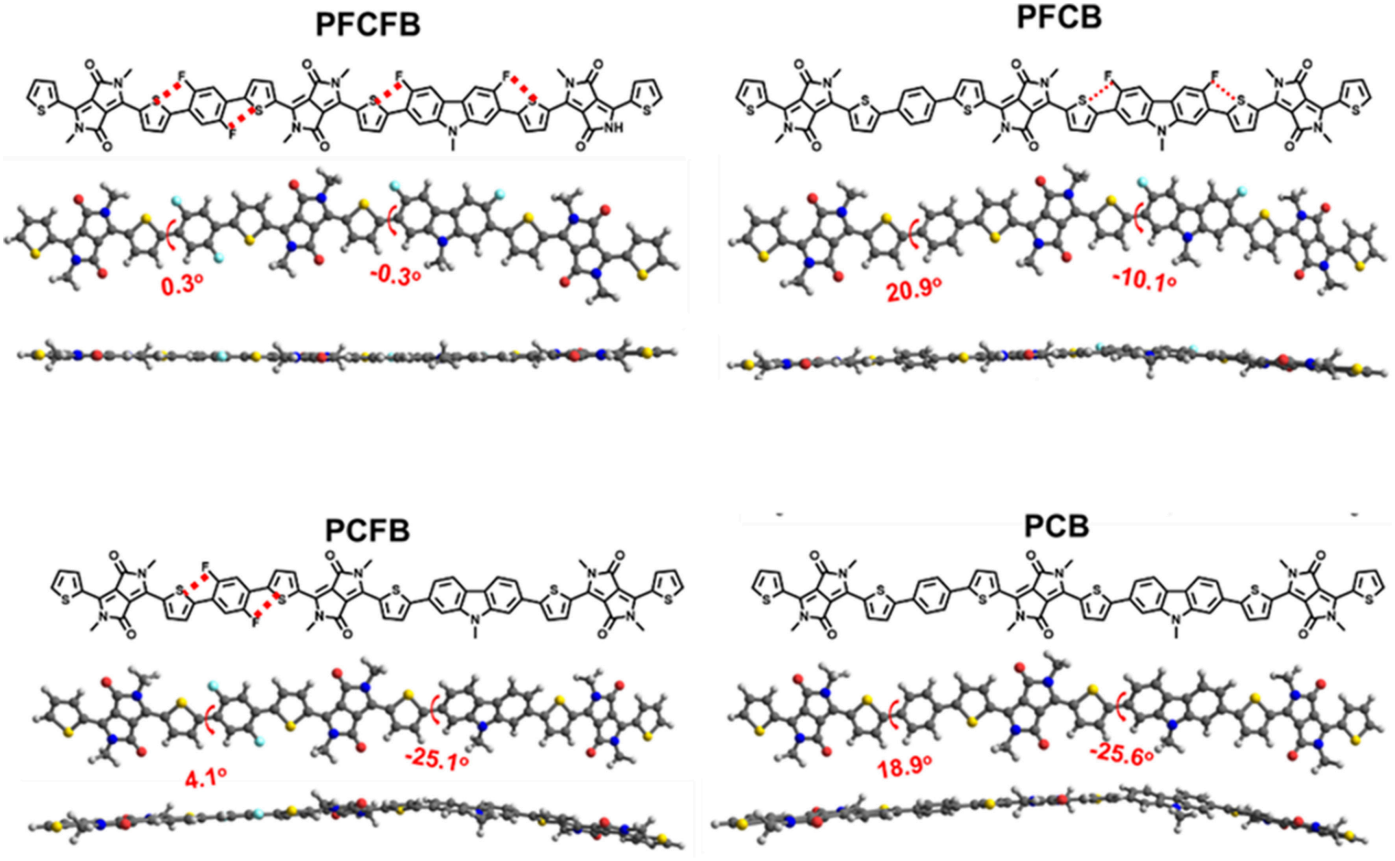
Optimized chemical geometries of the simplified repeating units using DFT at the B3LYP/6-31G(d) level.

Density functional theory (DFT) calculations at the B3LYP/6-31G(d) level was used to investigate the chemical geometry and electronic structures of the simplified repeating units. As shown in Figure 2, the two important dihedral angles in the repeating units are marked. The one formed by benzene and thiophene is significantly reduced from about 20° to only several degrees after fluorine substitution. However, the other one formed by carbazole and thiophene is quite large about 25° and only decreased to 10° after fluorine substitution. It is worth noting that these dihedral angles were approaching to zero degree after fluorination of both the carbazole and the benzene units, which endows excellent planarity of the repeating unit based on **PFCFB**. This planarity guarantees the outstanding transport mobility of the polymers which may give the high FF of the devices. **PCFB** is the other one who possesses a moderate planarity with two dihedral angles of 4.1° and 25.1°, which may give a reason why it has both a short lamellar and π-π stacking distance. Such an enhanced planarity of the aromatic fragments in fluorinated polymer chains arises from the intramolecular F···S interaction, which lock their planar conformations in the solid states (Jackson et al., 2013; Cheng et al., 2016). It is worth noting that the result of the calculations is consistent with the X-ray diffractions. As shown in Table S2, polymer film based on **PFCFB** exhibit the lowest d-spacing distance about 3.70 Å, the one based on **PCB** own a d-spacing distance of 3.76 Å, and the d-spacing distance of the **PFCB** and **PCFB** was between **PFCFB** and **PCB**. This phenomenon arises from the different planarity of the polymers caused by the differences in intramolecular non-covalent interactions. The electronic properties are also investigated *via* DFT calculations (see Figure S3). The tendency was consistent with CV measurements. The HOMO energy level moved downwards after the introduction of F atoms, which will be helpful to enhance *V*_oc_ of the devices based on fluorinated polymers.

## Photovoltaic Properties

**Figure 3:**
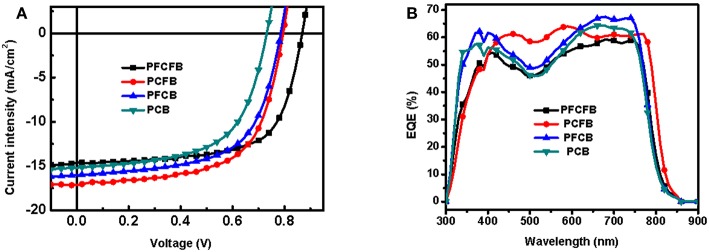
*J-V* **(A)** and EQE **(B)** curves of OSCs based on **PFCFB, PCFB, PFCB**, and **PCB**.

The photovoltaic properties of the polymers was investigated using conventional device with a structure of ITO/PEDOT:PSS/polymer:PC_71_BM/LiF/Al in which ITO and PEDOT:PSS mean indium tin oxide, poly(3,4-ethylenedioxythiophene):poly(styrenesulfonate), phenyl-C_71_-butyric acid methyl, respectively. Various conditions were employed to optimize the photovoltaic performance, such as different ratios of donor/acceptor, solvent additives and spin-coating rates. The optimized current-voltage (*J-V*) curves is shown in Figure 3. The active layer was spin-coated on the substrate with a solution of CHCl_3_ (with DIO as the additive). The optimized donor to acceptor weight ratio is 1:2 (w/w), and the optimized thickness is 100 nm by a spin-coating rate about 1,100 r/min with dilute polymer concentration of 3 mg/mL. The optimized photovoltaic parameters were summarized in Table 2. Devices based on **PFCFB** and **PCFB** gave the high PCE of 8.48 and 8.46%, respectively. It should be noted that PFCFB shows a different photovoltaic performance with PCDPP (Liu et al., 2016), which has a similar molecular structure as PFCFB. The difference comes from two aspects. First, PFCFB has a straight alkyl chain (n-octyl) on the carbazole unit; however, PCDPP has a branched one (2-ethylhexyl). Second, the processing solvents for depositing the active layer are different. The active layer was deposited from chlorobenzene solutions for PCDPP based device (Liu et al., 2016). In contrast, the active layer was deposited from a solution of CHCl_3_ for PFCFB. Here, the reason for using a solution of CHCl_3_ is that **PCFB** dissolves better in CHCl_3_ than in chlorobenzene. In order to make a comparison with the other three compounds, we thus chose chloroform as the processing solvent. The optimized **PFCB** and **PCB** based devices gave a relatively low PCE of 7.73 and 6.64%, respectively. Moreover, the *V*_oc_ of these devices was decreased with the reduction of F atoms due to the increase of HOMO energy level of polymers. Typically, the *V*_oc_ of PFCFB based device was 0.86 V which is among the highest *V*_oc_ of DPP-based polymers (Liu et al., 2016). The *V*_oc_ of **PCB** based device was decreased about 15% with a value of 0.73 V. The elevated *J*_sc_ of **PFCB** and **PCFB** based devices relative to that of **PCB** based one can be attributed to the reduced π-π stacking distance which facilitates the charge transport (vide infra). Although **PFCFB** demonstrated the highest *V*_oc_ among the four polymers, it shows the lowest *J*_sc_ value which arises from its low photoresponse in the wavelength range of 600–800 nm. External quantum efficiency (EQE) measurements were performed under monochromatic irradiation to investigate the spectral photoresponse and to verify the *J*_sc_ obtained from *J-V* curves. The photo-electron response region was contributed by both the two components of the blend film. In addition, the edge of EQE curves were mainly decided by the UV-vis absorption of the polymers due to the narrow absorption of the PC_71_BM. Therefore, devices based on **PCFB** exhibit the broadest photo-electron response region, while **PCB** based devices possess the narrowest one. It is worth noting that the *J*_sc_ obtained from EQE curves is within 5% deviation to that of *J-V* curves.

**Table 2:** Photovoltaic parameters of devices based on **PFCFC, PCFB, PFCB**, and **PCB**.

**Polymer**	**DIO**	***V_**oc**_*(V)**	***J_**sc**_*(mA/cm^**2**^)**	**FF**	**PCE (%)**
PFCFB	1%	0.86	14.60 (14.34)^a^	0.66	8.48
PCFB	0.8%	0.79	17.20 (16.56)^a^	0.62	8.46
PFCB	1%	0.78	16.21 (15.71)^a^	0.61	7.73
PCB	1%	0.73	15.19 (14.91)^a^	0.59	6.64

a *Calculated by EQE measurements*.

## Charge Transport Properties

The charge transport properties were characterized to further expose the differences in the four polymers. Hole and electron mobilities were measured by the space-charge-limited-current (SCLC) method with a structure of ITO/PEDOT:PSS/active layer/Au and ITO/ZnO/active layer/Al, respectively (shown in Figure S4). The hole and electron mobilities were listed in Table S3. The hole and electron mobility of **PCFB** was the highest one with a value of 4.63 × 10^−4^ and 2.40 × 10^−4^ cm^2^ V^−1^ s^−1^, respectively, which leads to the high *J*_sc_ of optimized device based on **PCFB**.

## Film Morphologies

**Figure 4:**
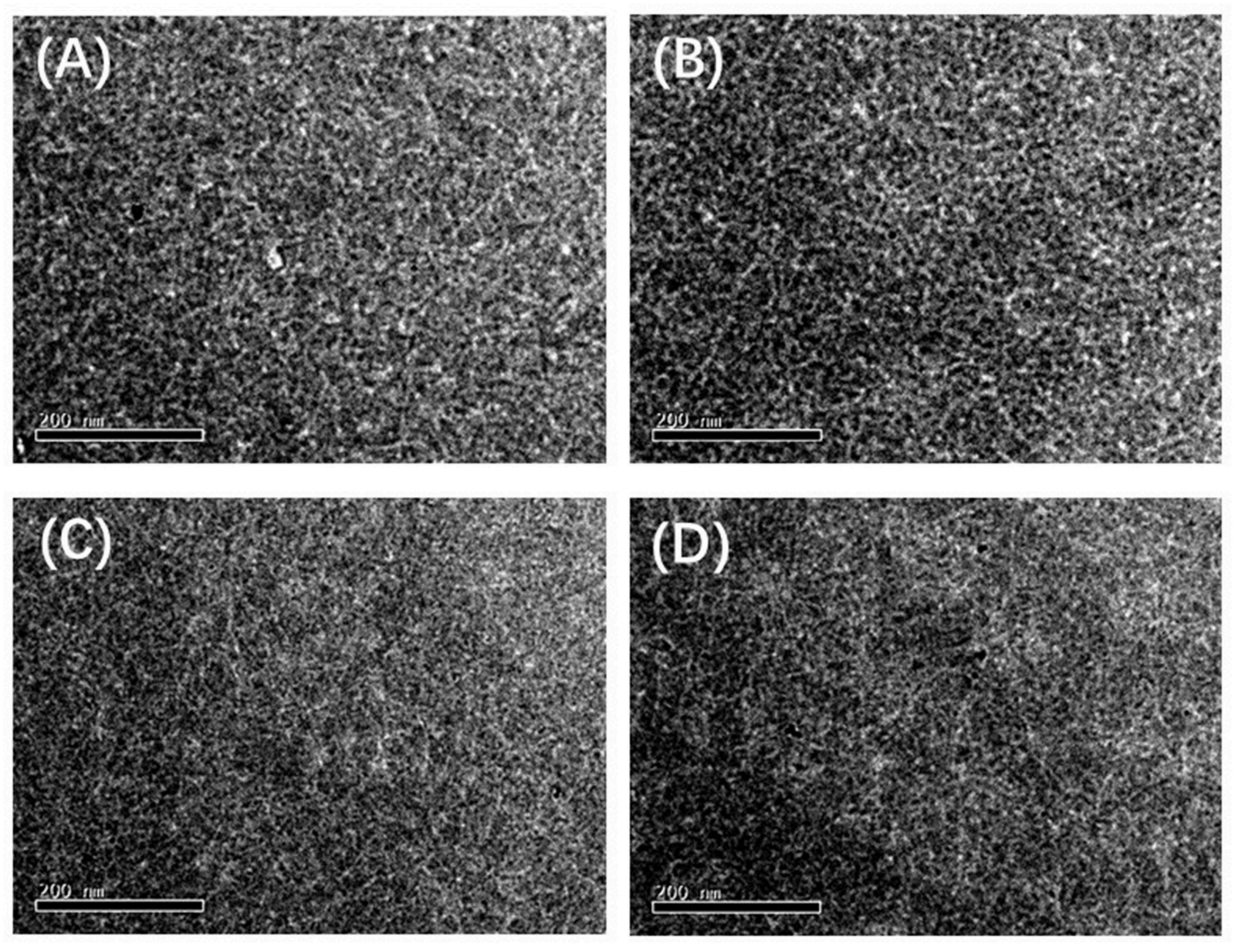
TEM images of the blend films based on the polymer **(A) PFCFB, (B) PCFB, (C) PFCB**, and **(D) PCB**.

In order to better understand the fluorine effect on the photovoltaic performance, the morphologies of the blend films were investigated by atomic force microscope (AFM) and transmission electron microscope (TEM). As shown in Figure S5, the corresponding root-mean-square (RMS) roughness (*R*_q_) values of **PFCFB**, **PCFB**, **PFCB**, and **PCB** based films are 1.51, 1.77, 1.41, and 1.26 nm, respectively. Networks of polymer fibrils can be found in the blended films (see Figure 4), which facilitate the charge separation and transport. Specially, proper aggregation was observed in Figure S5B based on **PCFB** which may explain why its hole and electron mobility is the highest and give the highest *J*_sc_.

## Conclusion

In summary, regioregular terpolymers by changing F atoms in the various moieties in the polymer chain were carefully designed and synthesized by Suzuki coupling. These polymers showed broad absorption in the visible region and exhibited a low bandgap <1.6 eV. From DFT calculations we know that the introduction of F atoms can flatten the molecular geometry between adjacent aromatic units due to the intramolecular supramolecular interaction. By introduction of F atoms to the benzene or carbazole segments or both of them, the molecular geometry can be modulated. Among the four terpolymers, **PFCFB** exhibited an excellent planarity with low dihedral angles. **PCFB** and **PFCB** showed a less good planarity than **PFCFB** but they are still much better than the unfluorinated one (**PCB)**. Their molecular geometries thus influence the corresponding UV-vis spectra and charge transport properties. The introduction of F atom into the polymer chains can also significantly affect their energy levels. We demonstrate that **PFCFB** based devices have the highest *V*_oc_ of 0.86 V which seriously reduce to 0.73 V after the remove of F atoms (the **PCB** case), while **PCFB** based ones give the highest *J*_sc_ of 17.20 mA/cm^2^. For these two polymers (**PFCFB** and **PCFB**), the highest PCE of ~8.5% was achieved. Our results give a clear explanation that how F atoms take effect on the regioregular terpolymers and modulate their photovoltaic performances. We show that selecting proper donor units with F atom to fabricate copolymers is an effective way to realizing high-efficiency polymer solar cells based on DPP units by enhancing in both *V*_oc_ and *J*_sc_.

## Data Availability

The raw data supporting the conclusions of this manuscript will be made available by the authors, without undue reservation, to any qualified researcher.

## Author Contributions

All authors listed have made a substantial, direct and intellectual contribution to the work, and approved it for publication.

### Conflict of Interest Statement

The authors declare that the research was conducted in the absence of any commercial or financial relationships that could be construed as a potential conflict of interest.

## Supplementary Material

Experimental details, instruments, OPV fabrication and measurements. XRD, TGA, SCLC measurements, DFT calculations, and AFM images.
