# Supplementary material for: Fluoro-Modulated Molecular Geometry in Diketopyrrolopyrrole-Based Low-Bandgap Copolymers for Tuning the Photovoltaic Performance
Source: Front Chem. 2019 May 15;7:333. doi: 10.3389/fchem.2019.00333 (PMC6530256; doi:10.3389/fchem.2019.00333)
Supplement: Supplementary file 1 [file Data_Sheet_1.docx]

Supplemental material for

**Fluoro-modulated Molecular Geometry in Diketopyrrolopyrrole-Based Low-Bandgap Copolymers for Tuning the Photovoltaic Performance**

**Cai’e Zhang, Yahui Liu, JiaTu, Shouli Ming, Xinjun Xu,* Zhishan Bo***

Beijing Key Laboratory of Energy Conversion and Storage Materials, College of Chemistry, Beijing Normal University, Beijing 100875, China.

**Experimental Part**

**Materials**

The original materials **1**, **2**, **3**, and **4** were synthesized as in reported literature (Du et al., 2013). PC_71_BM was purchased from Solarmer Materials Inc. Unless otherwise stated, all the chemicals were commercially available products and were used as received.

General synthesis of polymers:

A mixture of **1** (**2**) and **3** (**4**), NaHCO_3_, THF (20 mL), toluene (5 mL), and H_2_O (2 mL) was added to 100 mL Schlenk bottle and carefully degassed before and after Pd(PPh_3_)_4_ was added. After refluxed at 90 ^°^C for 3 days, phenylboronic acid and catalyst (Pd(PPh_3_)_4_) were added and refluxed for 4 h, and then bromobenzene and catalyst was added and refluxed to stop the polymerization. After that, 50 mL methanol was added to the reaction bottle and the polymer was filtered. The polymer was Soxhlet extracted with methanol, acetone, hexane dichloromethane and chloroform, then the residue was dissolved in boiling 1,1,2,2-tetrachloroethane (TCE) and precipitated into methanol, and the result solid was collected by filtration and dried under high vacuum. The dosages of the original materials were shown in **Table S1.**

**TABLE S1**| The dosages of the original materials.

| PFCFB | | PCFB | | PFCB | | PCB | |
| --- | --- | --- | --- | --- | --- | --- | --- |
| **1** | 139.04 mg | **1** | 125.80 mg | **2** | 99.28 mg | **2** | 91.73 mg |
| **3** | 44.66 mg | **4** | 37.84 mg | **3** | 32.55 mg | **4** | 28.16 mg |
| Pd(PPh_3_)_4_ | 4.53 mg | Pd(PPh_3_)_4_ | 4.22 mg | Pd(PPh_3_)_4_ | 3.89 mg | Pd(PPh_3_)_4_ | 3.65 mg |

**Instruments:**

UV-visible absorption spectra were obtained on a PerkinElmer UV-vis spectrometer (model Lambda 750). Atomic force microscopy (AFM) measurements were performed under ambient conditions using a Digital Instrument Multimode Nanoscope IIIA in the tapping mode. Transmission electron microscopy (TEM) images were obtained with a FEI Technai TF20 (Philip) transmission electron microscope. The thickness of the blend films was determined by a Dektak 6 M surface profilometer.

**OSC fabrication and measurements**

The device architecture was ITO/PEDOT:PSS/active layer/LiF/Al for solar cells. Pre-cleaned ITO substrates were treated by UV-ozone for 30 min. A thin layer of PEDOT:PSS was spin-coated on top of ITO substrate at 3500 rpm for 20 s and annealed at 150 °C for 20 min on a hotplate before being transferred into a glove box. Devices based on **PFCFB**, **PCFB**, **PFCB**, **PCB** as the electron donor and PC_71_BM as the electron acceptor were fabricated. The blend films keep the same optimal donor/acceptor weight ratio of 1:2 and deposited from solutions with the polymer concentration of 3 mg/mL in CHCl_3_ (**PFCFB**, **PFCB**, **PCB**: **with** 1% DIO added, and **PCFB** with 0.8% DIO added) by spin-coating at 1100 rpm for 50 s to obtain a film thickness of ~100 nm on the top of ITO/PEDOT:PSS. Finally, to complete the devices, the top electrode was thermally evaporated with a 4 Å LiF layer and followed by a 100 nm Al film at a base pressure below 10^−7^ Torr. Six cells were fabricated on one substrate with an effective area of 0.04 cm^2^. The measurement of the devices was conducted in a glove box without encapsulation. The temperature while measuring the *J*–*V* curves was approximately 25°C. *J*_sc_ values integrated from the EQE curves agreed well with those obtained from *J*–*V* tests (within 5% mismatch). The *J*–*V* curves were measured under AM 1.5G illumination at 100 mW cm^−2^ using an AAA solar simulator (XES-70S1, SAN-EI Electric Co., Ltd) calibrated with a standard photovoltaic cell equipped with a KG5 filter (certificated by the National Institute of Metrology) and a Keithley 2400 source-measure unit. The EQE data were obtained using a solar cell spectral response measurement system (QER3011, Enli Technology Co. Ltd), and the intensity was calibrated with a standard single-crystal Si photovoltaic cell.

**FIGURE S1**| X-ray diffraction patterns of polymer thin films.

**TABLE S2**| Diffraction peaks and distance of the polymers measured by the X-ray diffractions.

| Polymer | 2θ (°) | 2θ (°) | π-π distance (Å) |
| --- | --- | --- | --- |
| **PFCFB** | 4.76 | 24.03 | 3.70 |
| **PCFB** | 4.84 | 23.85 | 3.73 |
| **PFCB** | 4.75 | 23.92 | 3.72 |
| **PCB** | 4.79 | 23.63 | 3.77 |

**FIGUER S2**| Thermogravimetric analysis (TGA) curves of polymers.


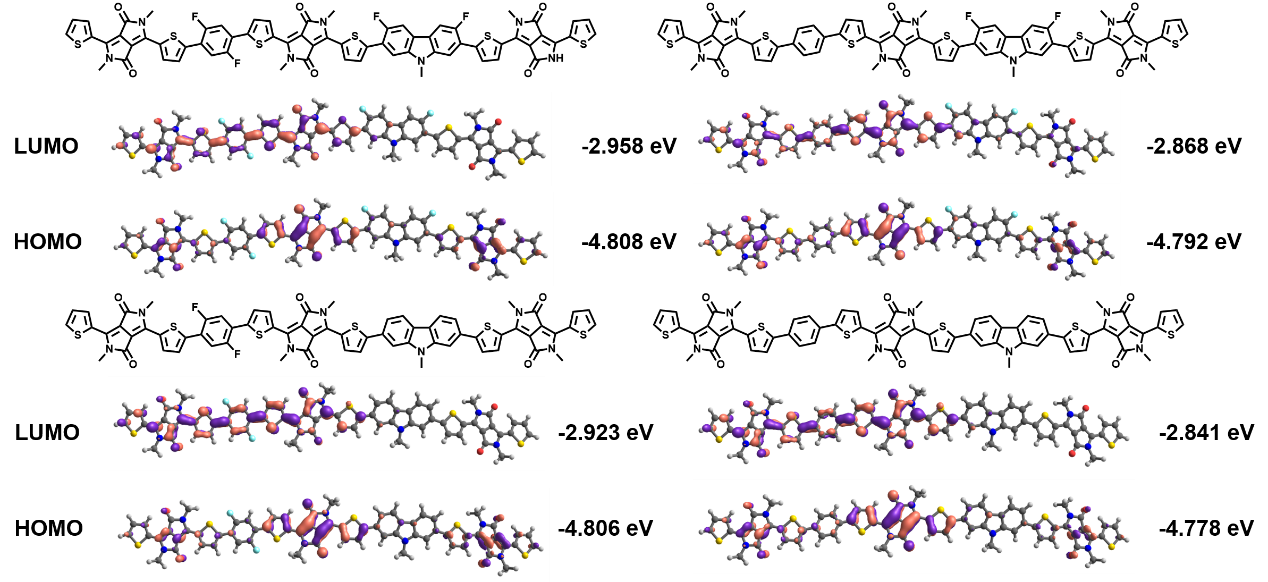


**FIGURE S3**|. Electronic properties of the simplified monomers *via* DFT calculations.

**FIGURE S4**| *J^1/2^*-*V* curves for measuring the electron (A) and hole (B) mobility of blend films.

**TABLE S3.** Hole and electron mobilities of devices based on **PFCFC**, **PCFB**, **PFCB** and **PCB**.

| Polymer | *μ*_e_ (× 10^-4^ cm^2^ V^-1^ s^-1^) | *μ*_h_ (× 10^-4^ cm^2^ V^-1^ s^-1^) |
| --- | --- | --- |
| **PFCFB** | 2.61 | 1.16 |
| **PCFB** | 4.63 | 2.40 |
| **PFCB** | 3.60 | 2.06 |
| **PCB** | 2.88 | 1.89 |


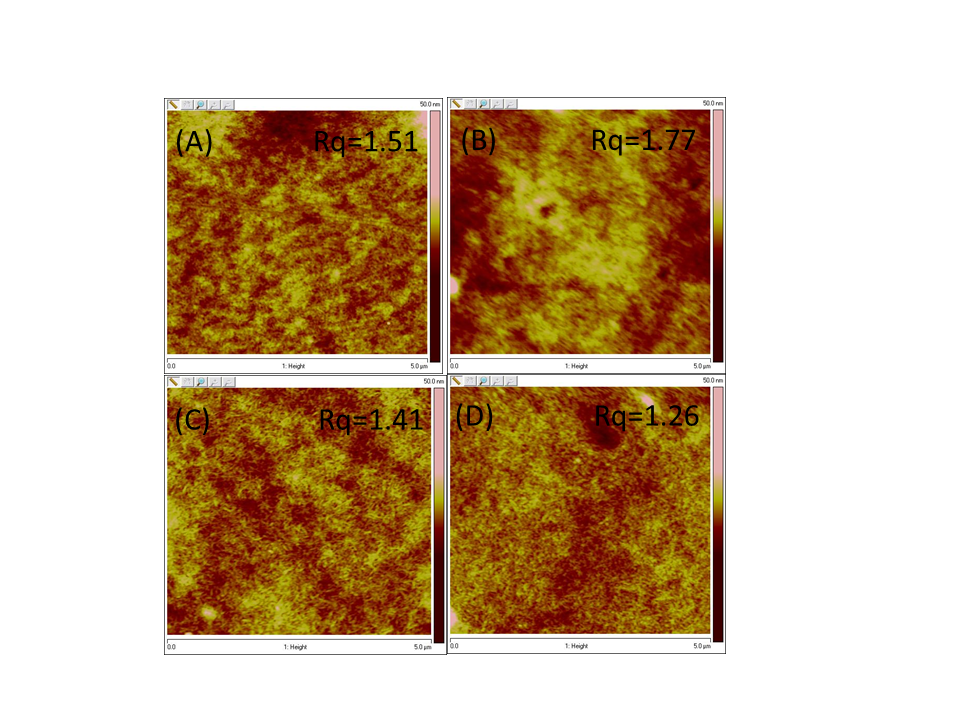


**FIGURE S5**| AFM graphs of the blend films based on the polymers (A) PFCFB, (B) PCFB, (C) PFCB, and (D) PCB (size: 5 μm × 5 μm).

**References**

Du, C., Li, W., Duan, Y., Li, C., Dong, H., Zhu, J., et al. (2013). Conjugated polymers with 2,7-linked 3,6- difluorocarbazole as donor unit for high efficiency polymer solar cells. *Polym. Chem.* 4(9)**,** 2773. doi: 10.1039/c3py00177f.
